# Microbial Dynamics on Different Microplastics in Coastal Urban Aquatic Ecosystems: The Critical Roles of Extracellular Polymeric Substances

**DOI:** 10.1021/acs.est.5c03796

**Published:** 2025-05-20

**Authors:** Cuijie Feng, Ziyan Liang, Xin Liao, Kairong Lin, Yujia Zhai, Gang Liu, Francesca Malpei, Anyi Hu

**Affiliations:** † Center for Water Resources and Environment, School of Civil Engineering, 26469 Sun Yat-sen University, Guangzhou 510275, PR China; ‡ CAS Key Laboratory of Urban Pollutant Conversion, 85406 Institute of Urban Environment, Chinese Academy of Science, Xiamen 361021, PR China; § State Key Joint Laboratory of Environment Simulation and Pollution Control, School of Environment, 47836 Beijing Normal University, Beijing 100875, PR China; ∥ Key Lab of Aquatic Chemistry, State Key Lab of Regional Environment, Research Centre for Eco-Environmental Sciences, Chinese Academy of Sciences, Beijing 100085, China; ⊥ Sanitary engineering, Department of Water Management, 2860 Delft University of Technology, Delft 2628 CN, The Netherlands; # Department of Civil and Environmental Engineering, Politecnico di Milano, Milan 20133, Italy; ∇ Carbon Neutral Innovation Research Center and Fujian Key Laboratory of Marine Carbon Sequestration, Xiamen University, Xiamen 361105, PR China

**Keywords:** microplastics (MPs), microbial community, temporal succession, extracellular polymeric substances (EPS), metagenomic sequencing

## Abstract

Microplastics (MPs) serve as carriers for microbial community colonization, forming unique ecosystems known as plastispheres in urban aquatic ecosystems. However, interactions among microbes, extracellular polymeric substances (EPS), and MPs remain poorly understood. This study investigates microbial consortia and their EPS secretion behaviors across various plastispheres at two representative coastal urban water sites. Permutational multivariate analysis of variance revealed that MP type significantly influenced microbial community structures in reservoir environments (*R* ^2^ = 0.60, *p* < 0.001), highlighting the pronounced impact of MP types in high-quality urban waters. Specific microbial phyla and genera were identified as key contributors to EPS compositional variations across different plastispheres. Hierarchical partitioning results identified Acidobacteria, Nitrospirae, and Planctomycetes as influential phyla positively affecting EPS composition. Spearman correlation analysis pinpointed *Robiginitialea* (positive correlation) and *Fimbriiglobus* (negative correlation) as critical genera influencing EPS dynamics. Moreover, EPS-related gene abundance corresponded closely with observed EPS compositional differences. Dominant genes associated with protein biosynthesis included *xapD* in reservoirs and *glnA* in bays, while *glmS* and *eno* were predominant for polysaccharide biosynthesis in bays. This research advances our understanding of microbial-EPS-MP interactions in urban water systems, offering critical insights into ecological remediation and risk assessment of MP pollution.

## Graphical abstract

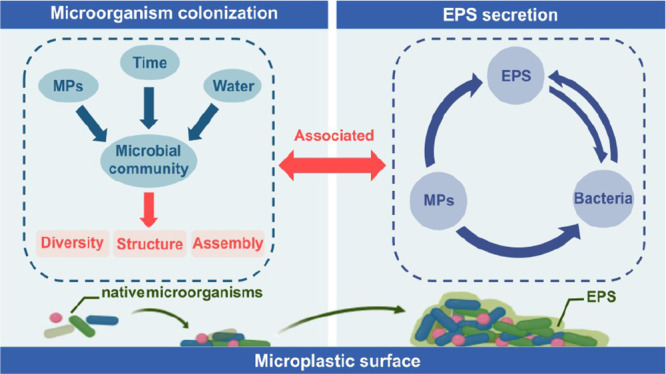

## Introduction

With the development of society and the increase of industrial production, microplastics (MPs), defined as plastic particles smaller than 5 mm, have emerged as a widespread pollutant.^1^ MPs are extensively dispersed across diverse aquatic ecosystems, including urban rivers, lakes, reservoirs, and bays.^2^ Many studies have reported high abundances of polyurethane (PE) and polypropylene (PP) in these environments, followed by polystyrene (PS) and polyvinyl chloride (PVC).^3–5^ Tire material (TM) particles have also been identified as a notable contributor.^6^ The high prevalence of MPs has led to significant environmental issues in aquatic environments.

The colonization of microorganisms on MPs warrants greater attention due to the environmental challenges posed by these pollutants. MPs possess a large specific surface area, high porosity, and a strong ability to adsorb water pollutants.^7^ These properties make MPs effective carriers for microorganisms, providing a stable habitat that forms a unique ecological niche referred to as the “plastisphere”.^8^ Recent research has explored the dynamic succession, assembly processes, and functional characteristics of microbial communities on MPs. For instance, Wang et al. demonstrated that the bacterial community structure on MPs was influenced by both temporal and spatial variations, with significant differences observed in bacterial composition across different types of MPs.^9^ Zhang et al. investigated the effects of exposure time, locations, and MP types, concluding that the exposure time played a crucial role in shaping bacterial community composition. Their findings revealed that Chao1 index of prokaryotic communities increased with prolonged exposure, and the community assembly was primarily driven by the homogenization in freshwater lakes.^10^ However, current studies focused on different locations within the same urban aquatic ecosystem, lacking horizontal comparisons across distinct urban aquatic ecosystems. Moreover, these studies predominantly examined abiotic factors affecting microbial community structure, while neglecting the influence of biotic factors, such as native microorganisms. Consequently, a more comprehensive understanding of the processes governing microbial communities’ formation on MPs in urban aquatic ecosystems is essential.

Extracellular polymeric substances (EPS) play an important role in the microbial colonization process on MPs. EPS primarily consist of biomolecules, such as proteins, polysaccharides, humic acids, and lipids, which provides a stable framework that enhances the attachment of microorganisms to the surface of MPs.^11^ Recently, increasing attention has been directed toward understanding EPS secretion on MPs in urban aquatic ecosystems. Gong et al. demonstrated that 5 μm MPs enhanced the secretion of protein-rich EPS,^12^ while Huang and co-workers found that 100–300 mg L^–1^ PE significantly promoted EPS production, specifically humic acids.^13^ Despite these findings, limited research has explored the relationship between EPS secretion and microbial communities within the plastisphere. Bridging this knowledge gap is a key focus of this study.

Based on the above information, it can be inferred that both MP types and the characteristics of urban aquatic ecosystems play a significant role in influencing the dynamic succession of microbial communities and their EPS secretion capacity. To test this hypothesis, five common MPs (i.e., TM, PS, PE, PP, and PVC) were exposed in situ to two distinct water environments (freshwater and brackish water) for 90 days. The study aimed to elucidate the bacterial succession patterns at the temporal scale and assembly patterns of the microbial communities on MPs across different urban aquatic ecosystems. This study delved into the EPS secretion dynamics during bacterial colonization on MPs, establishing critical links between “EPS-microbial communities” and “EPS-biological functions”.

## Materials and Methods

### Research Site, Sampling, and Environmental Parameter Measurement

Field experiments were conducted in Zhuhai, China, to investigate microbial–microplastic interactions in aquatic environments. Two representative sites were selected: a freshwater reservoir and a brackish bay. To characterize environmental conditions, key water quality parameters (pH, conductivity, dissolved oxygen, temperature, and salinity) were measured using a portable analyzer (HQ4300, HACH, USA). Nutrient levels (ammonia, nitrite, nitrate, and phosphate), which influence microbial activity, were determined following standard protocols.^14^ Compared with the bay, the reservoir had higher pH and dissolved oxygen, lower salinity, and reduced nutrient concentrations. Detailed water quality data are presented in Text S6 and Figure S1.

### Microplastic Incubation Experiment Setup

Microplastic (MP) types used included ground tire rubber (TM), polyethylene (PE), polypropylene (PP), polystyrene (PS), polyvinyl chloride (PVC), and biodegradable plastic (GB), all ∼1 mm in diameter. TM was prepared from tires; other MPs were sourced from Hongxing Polymer Materials Co., Ltd. (Dongguan, China). For each MP type, 3 g was placed in 40-mesh nylon bags and secured in mesh cages deployed at both sites (Figure S1). Samples were retrieved on Days 0, 15, 30, 60, and 90, then stored at −20 °C for EPS and DNA analysis. Triplicate sample sets were collected at each site.

### EPS Extraction and Composition Analysis

EPS was extracted using a modified heat–Na_2_CO_3_ method.^15^ Briefly, 1 g of freeze-dried sample was mixed with 30 mL of extraction buffer (0.5% Na_2_CO_3_, 0.6% NaCl) in centrifuge tubes, heated at 80 °C for 30 min, and centrifuged at 10,000 g for 15 min. Supernatants were collected, filtered through 0.45 μm PES filters (JINTENG, China), and stored at −20 °C. Protein content was quantified using the Bicinchoninic Acid Kit (Sigma-Aldrich, USA), polysaccharides via the phenol-sulfuric acid method,^16^ and humic substances with a modified Folin–Lowry assay.^17^

### DNA Extraction, 16S rDNA Amplicon, and Metagenomic Shotgun Sequencing

DNA was extracted using the FastDNA SPIN Kit for Soil (Qbiogene-MP Biomedicals, USA), with 0.3 g of each sample processed in duplicate. Sequencing was conducted by Novogene (Tianjin, China). For 16S rDNA amplicon sequencing, the V4–V5 region was amplified using primers 515F (5′-GTGYCAGCMGCCGCGGTA-3′) and 907R (5′-CCGYCAATTYMTTTRAGTTT-3′). PCR products were purified (QIAquick Kit, Qiagen, USA), and analyzed using LOTUs2. High-quality reads (>100 bp, quality score > 25, homopolymers ≤ 6 bp) were retained.

For metagenomics, ∼30 μL of DNA was used to construct libraries with 350 bp inserts. Sequencing was performed on the Illumina HiSeq 6000 platform (2 × 150 bp), generating ∼15 GB of raw data per sample. Clean reads were obtained using KneadData v0.6.1 (Trimmomatic: “SLIDINGWINDOW:4:20 MINLEN:50”; Bowtie2: “very-sensitive”). Contigs ≥ 500 bp were assembled with MEGAHIT v1.2.9.^18^ ORFs were predicted using Prodigal v2.6.3 (-p meta).^19^ Taxonomic classification and abundance estimation were performed with Kraken2 v2.0.7 and Bracken v2.0.^20,21^ Functional profiling used METABOLIC v4.0, aligning ORFs to the KEGG database.^22^ Contig abundance was normalized to RPKM using CoverM (github.com/wwood/CoverM). Sequencing data are available under NCBI BioProject IDs PRJNA1141234 and PRJNA1215273.

Microbial α-diversity was assessed using Kruskal–Wallis ANOVA in SPSS 27, with Shannon and Chao1 indices calculated using the *vegan* package in R 4.4.0.^23^ β-diversity was analyzed via NMDS using Bray–Curtis distances, and PERMANOVA (ADONIS) tested community differences. Community assembly processes (e.g., selection, dispersal, drift) were evaluated using the *iCAMP* package.^24^ Visualizations were produced with Origin 2024.

### Statistical Analysis

Variation partitioning analysis (VPA) using *vegan* identified the influence of environmental variables, microbial colonization, and exposure time on microbial abundance and EPS composition. Hierarchical partitioning (HP) was conducted using *rdacca.hp* to quantify the contribution of individual factors.^25^ Spearman correlation (SPSS 27) explored links between microbial taxa and EPS components. Plots were generated in Origin 2024 and Cytoscape. Statistical significance was set at *p* < 0.05.

### Water Quality at Each Research Site

As shown in Figure S1a–d, both sites were weakly alkaline, with the reservoir exhibiting consistently higher pH. Dissolved oxygen in the reservoir declined over time but remained above bay levels. Salinity in the reservoir was stable (<0.05‰), while the bay reached 9.31‰ by Day 90, confirming its brackish nature. The bay also exhibited higher nutrient levels: ammonia peaked with a 1.008 mg L^–1^ difference compared to the reservoir; nitrite remained below 0.1 mg L^–1^; nitrate in the reservoir briefly exceeded that in the bay on Day 60; phosphate in the bay was generally 0.1–0.2 mg L^–1^, with minimal fluctuation in the reservoir.

## Results

### Microbial Community Structures and Diversities

Microbial diversity patterns demonstrated clear environmental and MP stratification. The Shannon and Chao1 indices (Figure 1a,b) revealed MP biofilms maintained elevated α-diversity compared to planktonic communities throughout exposure. Freshwater MP consortia exhibited MPs-dependent properties – PS supported maximum Shannon diversity while TM hosted peak Chao1 richness, suggesting differential habitat specialization (Table S1). Brackish systems showed diminished α-diversity contrast between MPs and water, though MP biofilms retained marginally higher values, potentially indicating greater environmental filtering in marine systems. β-diversity analysis including nonmetric multidimensional scaling (NMDS) and permutational multivariate analysis of variance (PERMANOVA) revealed different ecological drivers: reservoir communities showed strong MP clustering (*R* ^2^ = 0.60, *p* < 0.001) with PVC forming distinct niche (Figure S2a), while brackish systems exhibited temporal succession dominance (*R* ^2^ = 0.44, *p* < 0.001) with late-stage MP convergence (Figure S2b).

**Figure 1:**
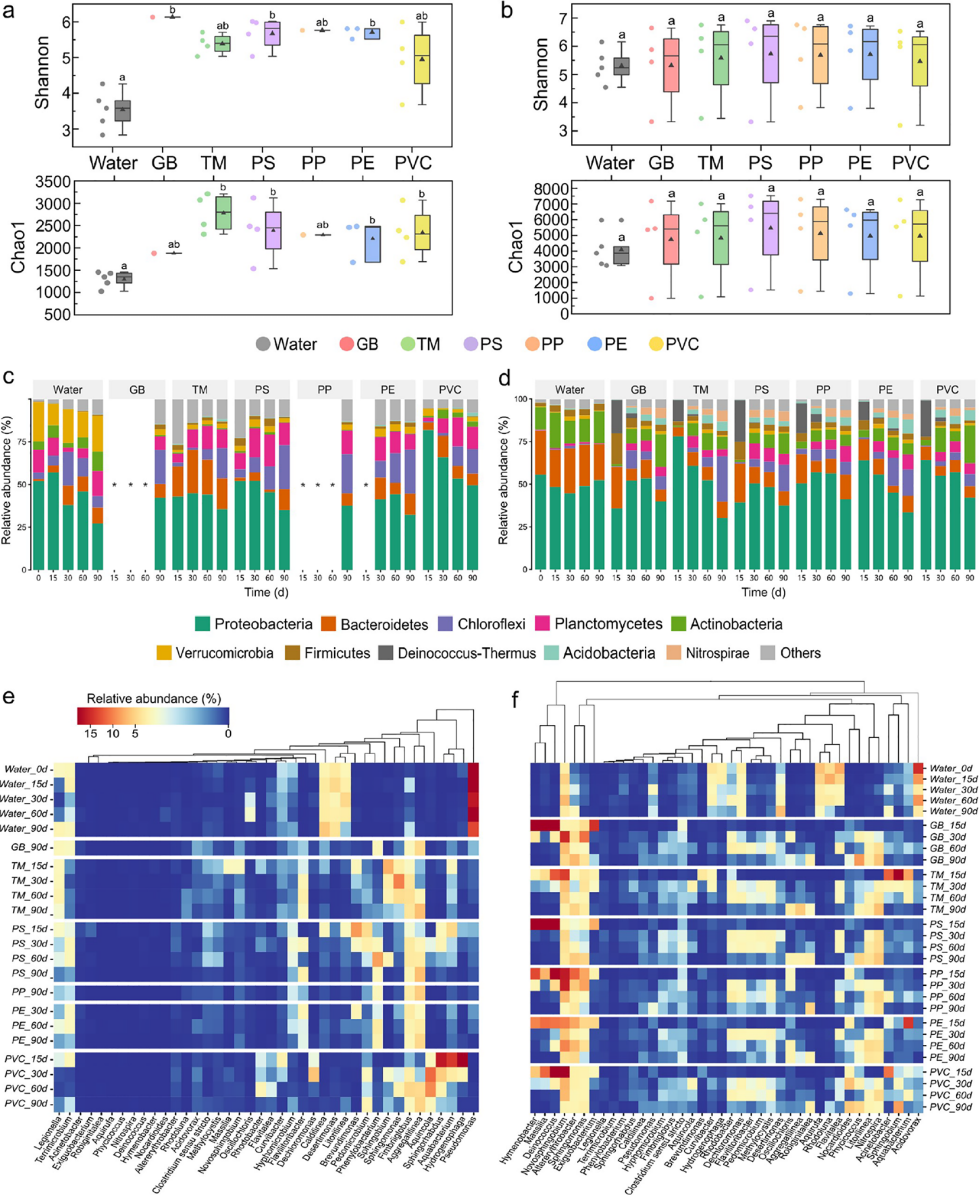
Comparative analysis of microbial community structure and diversity across aquatic environments. (a-b) biodiversity metrics: (a) Reservoir α-diversity indices; (b) Bay α-diversity indices. (c,d) Phylum-level taxonomic stacking diagrams, (e,f) heatmaps of top 40 genera: (c,e) Freshwater reservoir communities; (d,f) Brackish bay communities. ▲: mean value. Δ: statistical outlier. Missing reservoir data labeled with “*”: Excluded due to insufficient microbial biomass (<10 ng μL^–1^ DNA). PERMANOVA: *** *p* < 0.001.

Figure 1c,d presents the phylum-level taxonomic composition of microbial communities in two aquatic environments. In the freshwater reservoir, Proteobacteria (27.2–57.0%) and Verrucomicrobiota (12.7–23.0%) constituted the dominant phyla in water column communities. MP biofilms exhibited distinct colonization patterns, with Proteobacteria dominant (32.2–81.9%), alongside high relative abundances of Bacteroidetes (1.5–25.4%), Chloroflexi (0.6–26.0%), and Planctomycetes (2.3–18.7%). Temporal analysis revealed dynamic succession patterns: PVC-associated Proteobacteria peaked at 81.9% by Day 15 before declining to 49.5% on Day 90, while Chloroflexi demonstrated progressive enrichment on glass beads (GB), PS, PP, and PE, exceeding 20% abundance by Day 90. Niche-specific colonization was evident as Bacteroidetes preferentially accumulated on TM substrates (17.6–25.4%), whereas Verrucomicrobiota and Actinobacteria exhibited notable depletion relative to planktonic communities. Brackish bay ecosystems maintained Proteobacteria dominance (44.7–55.7% in water; 30.3–78.2% on MPs), though with different secondary colonizers. Water communities featured Bacteroidetes (21.0–26.3%) and Actinobacteria (13.2–20.8%), while microbial communities on MP biofilms reshaped over time. By the terminal exposure stage, MPs-dependent specific phylum succession emerged: Actinobacteria predominated on PVC, while Chloroflexi dominated TM, PS, and PE surfaces. Early colonizers including Deinococcus-Thermus (ubiquitous across MPs) and Firmicutes (GB/PS-specific) exhibited transient dominance, contrasting with the progressive enrichment of Planctomycetes, Acidobacteria, and Nitrospirae throughout the colonization period.

Genus-level analysis revealed distinct colonization patterns between planktonic and MP-associated communities (Figure 1e,f). In reservoir ecosystems, water communities were dominated by *Pseudomonas* (13.4–38.3%), *Litorilinea*, *Desertimonas*, and *Caldilinea*, while MP biofilms showed taxonomic specialization with *Aggregatilinea* and *Fimbriiglobus* as core colonizers. Temporal progression revealed *Aggregatilinea*’s competitive dominance through sustained enrichment, contrasted by *Brevundimonas*’s transient colonization (TM/PS/PE) showing rapid decline after initial establishment. MPs-specific preferences emerged: *Sphingomonas*/*Sphingobium* exhibited TM specialization, while *Phenylobacterium* demonstrated PS/PP/PE affinity. PVC surfaces hosted unique consortia (*Dechloromonas*, *Aquincola*, *Hydrogenophaga*, *Sphingorhabdus*, *Aquabacterium*, *Rhodobacter*) that underwent progressive succession despite initial dominance. Brackish bay communities displayed environmental filtering, with water communities’ dominance by *Acidovorax* (3.1–14.9%), *Flavitalea*, *Rhodoluna, Aquirufa*, and *Novosphingobium*. Remarkably, only *Desertimonas* maintained aquatic environment prevalence, while *Novosphingobium* emerged as a versatile MP colonizer. Early successional taxa (*Hymenobacter*, *Massilia*, *Deinococcus*, *Novosphingobium*, *Sphingomonas*, and *Exiguobacterium*) displayed time-dependent displacement, making way for late-colonizing specialists (*Robiginitialea*, *Phycicoccus*, *Litorilinea*, and *Nitrospira*) that established during prolonged exposure.

### Determinants of Microbial Community Assembly on Microplastics

Null model analysis showed that both stochastic (dispersal limitation, homogenizing dispersal, and drift) and deterministic (heterogeneous selection and homogeneous selection) processes mediated community assemblies, but distinct ecological drivers governing MP biofilm assembly across different environments (Figure 2a,b). In freshwater reservoirs, deterministic processes predominated with homogeneous selection accounting for 38.2% of community assembly, reflecting strong environmental filtering. In contrast, brackish systems exhibited stochastic dominance (Σ = 58.7%) driven by dispersal limitation (45.3%), suggesting reduced niche specialization. Variation partitioning analysis (VPA) demonstrated 91.2% of community variance through biotic-abiotic factors (Figure 2c). The influence order followed: native microorganisms (12.0% pure effect) > temporal progression > physicochemical parameters > nutrient availability.

**Figure 2:**
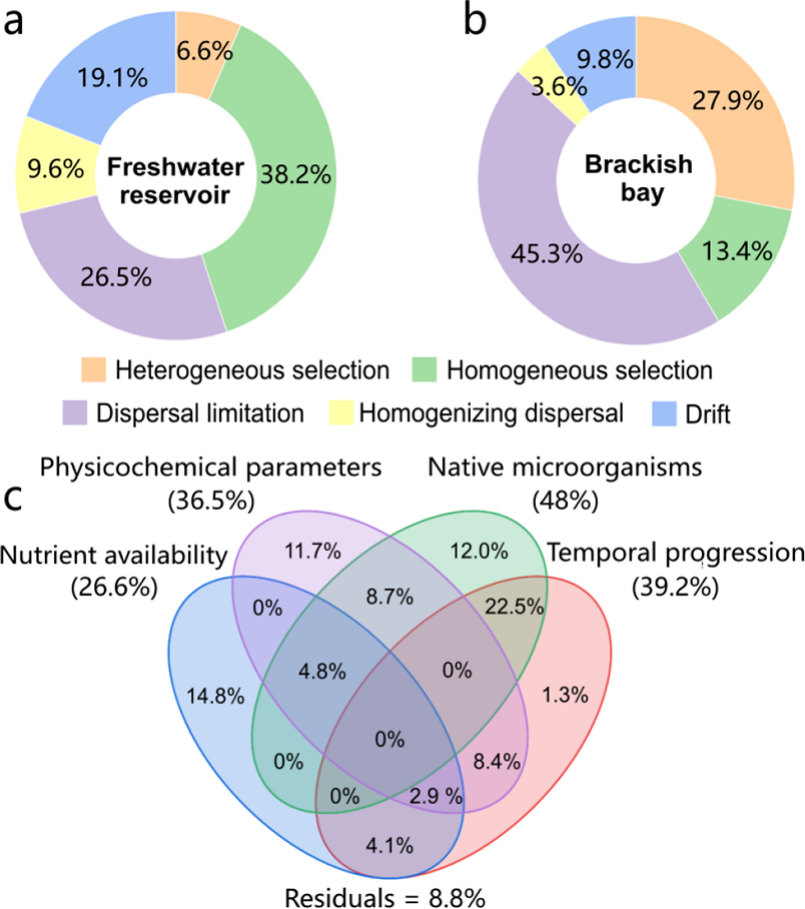
Community assemblies on MPs in (a) the freshwater reservoir and (b) the brackish bay. (c) VPA for explaining the relative abundance of microorganisms with explanatory variables (nutrient availability, physicochemical parameters, native microorganisms, and temporal progression).

### Changes in EPS Components on MPs

EPS components including proteins, polysaccharides, and humic acids on MPs were analyzed (Figure 3a,b). Significant spatial and temporal patterns in EPS dynamics were observed. EPS concentrations were consistently higher in the bay compared to the reservoir, with humic acids showing the most pronounced increase. Temporal trends revealed a consistent increase in EPS components and protein-to-polysaccharide ratios on MPs over time, highlighting a strong temporal regulation of microbial activity. MP type had a notable impact on EPS secretion in conjunction with spatiotemporal dynamics. In the reservoir, EPS secretion peaked on TM and was lowest on PS. Specifically, protein secretion was weak on PS and PP, while polysaccharide secretion remained stable on TM and PE, but more active on PP. In both environments, humic acid secretion on MPs was generally low. In the bay, temporal EPS dynamics were characterized by an initial increase followed by a decline on TM and PS. Across all MPs, PS consistently exhibited the lowest EPS secretion, while PP and PE supported the highest concentrations. By Day 90, EPS concentrations on PP of proteins, polysaccharides, and humic acids respectively reached 68.8 ± 3.2, 110.8 ± 1.3, and 62.2 ± 3.3 mg L^–1^, while those on PE reached 78.9 ± 8.4, 95.4 ± 7.2, and 50.4 ± 1.0 mg L^–1^, respectively. Protein-to-polysaccharide ratios were higher on TM and PVC in the reservoir, (exceeding 1.0 for PVC), with PVC in the bay reaching a ratio of 1.5 on Day 90. Differences among other MPs were negligible.

**Figure 3:**
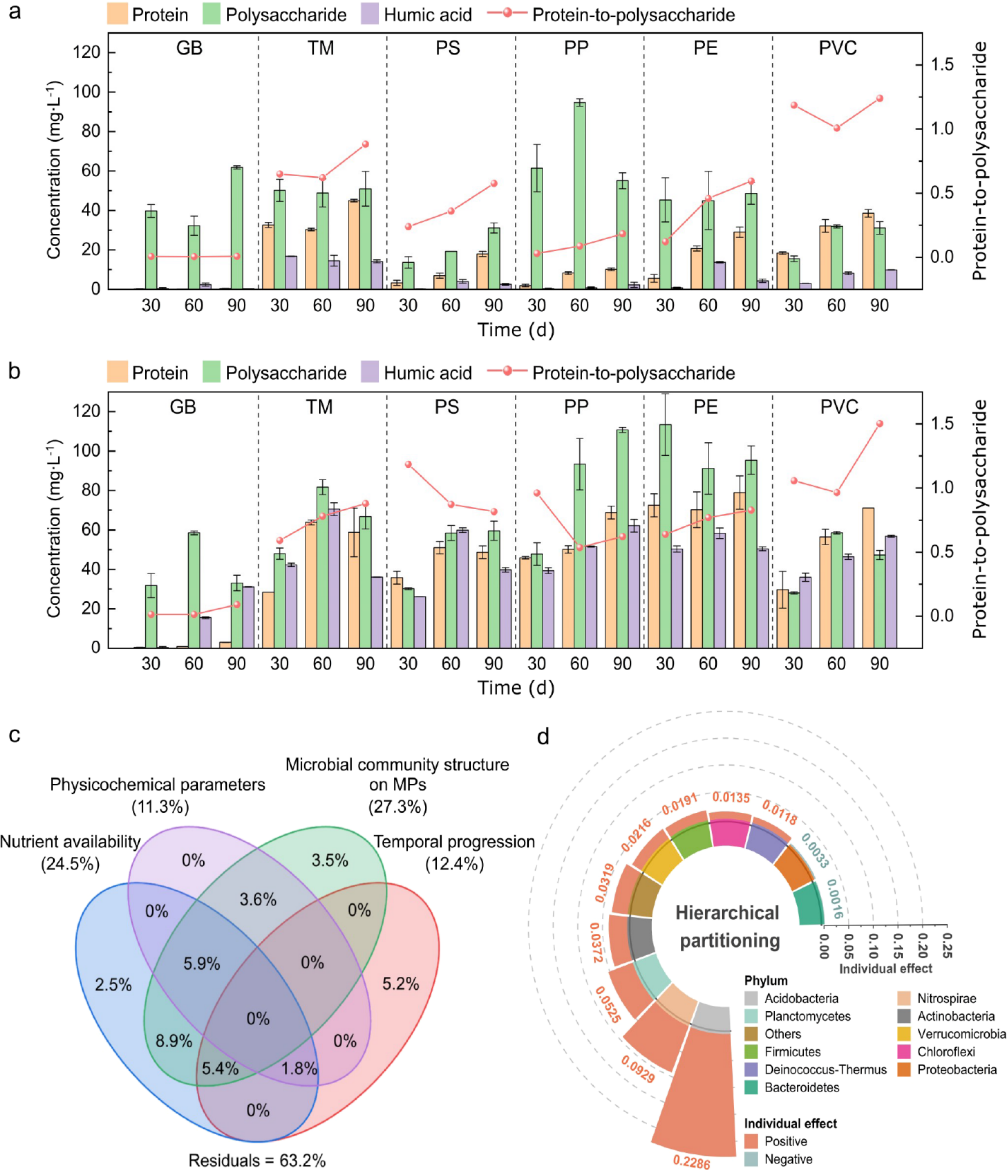
Dynamics of EPS on MPs across aquatic environments: (a) Freshwater reservoir and (b) Brackish bay. (c) VPA quantifying ecological drivers of EPS variance. (d) Phylum-level contributions to EPS secretion through hierarchical partitioning analysis.

VPA attributed 41.3% of the variance in EPS concentrations on MPs to biotic and abiotic factors (Figure 3c). Microbial community structure was the dominant driver, explaining 27.3% of the variance. Furthermore, hierarchical partitioning analysis (Figure 3d) revealed that most phyla exerted positive effects on EPS concentration variation. Acidobacteria (0.23), Nitrospirae (0.09), and Planctomycetes (0.05) emerged as the major contributors, indicating their significant roles in EPS regulation on MPs.

### Genus-Specific Associations with EPS Secretion

Spearman correlation analysis revealed distinct phylogenetic patterns in EPS regulation across MP substrates (Figure 4a). Thirty-three genera demonstrated significant EPS correlations (*p* < 0.05), categorized as (1) Unidirectional enhancers (only positive correlations); (2) EPS suppressors (only negative correlations); (3) MP-dependent regulators (positive/negative correlations). Network analysis (Figure 4b) identified *Robiginitialea* as the primary EPS promoter (degree = 9) contrasting with *Fimbriiglobus*’s inhibitory role (degree = 8). Substrate specialization emerged strongly: polysaccharide-associated taxa preferentially colonized TM (degree = 15); humic acid dynamics showed broader phylogenetic linkages (PVC/PE/PS/TM: > 10 genera); Protein correlations exhibited limited taxonomic breadth (<10 genera on each MP). It is noteworthy that correlations between some genera (e.g., *Oscillochloris*, *Sphingorhabdus*, and *Aquincola*) and EPS components differed on different MPs.

**Figure 4:**
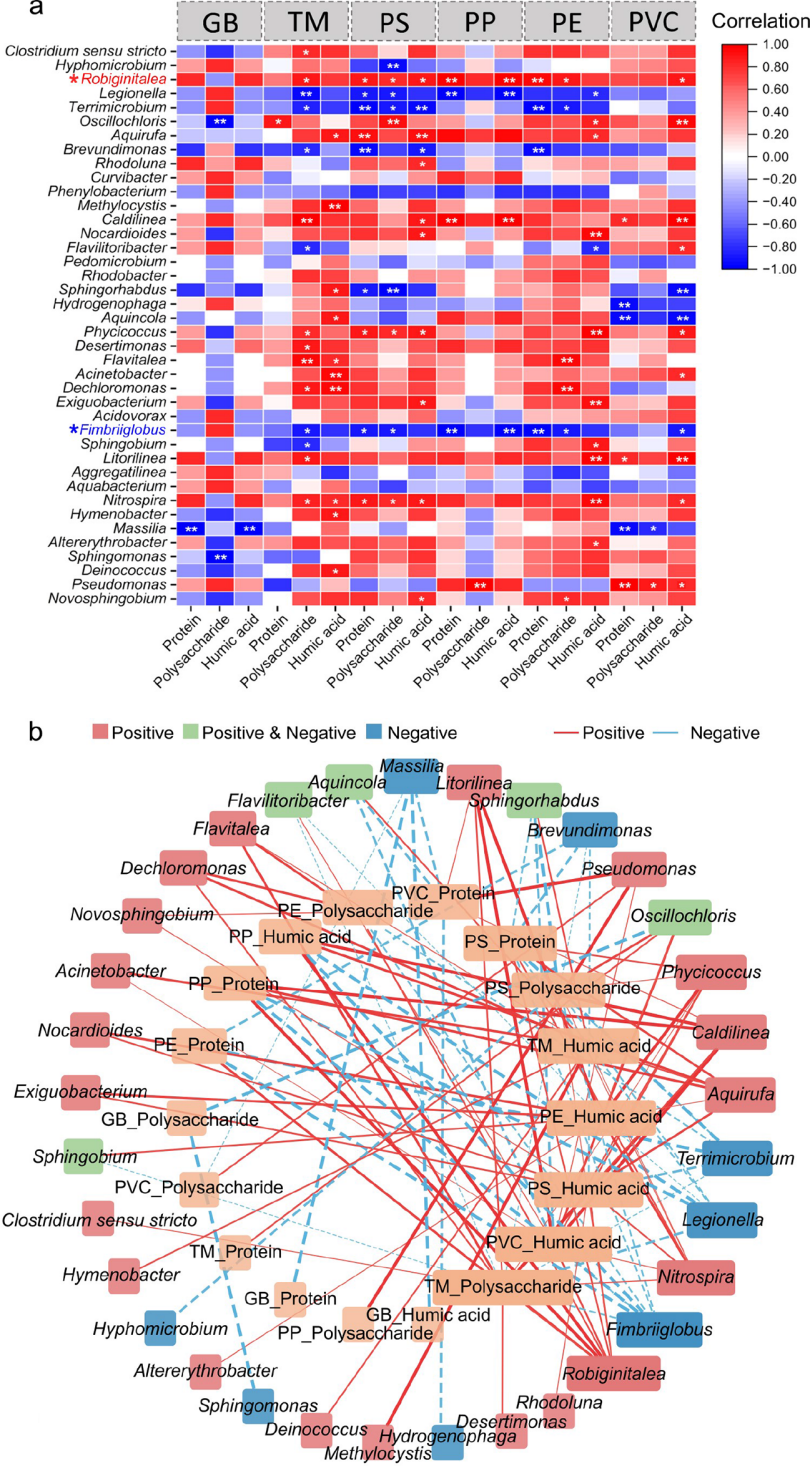
Genus-level associations between EPS components and microbial taxa. (a) Heatmap of Spearman correlations (ρ). Significant relationships (**p < 0.01, *p < 0.05, |ρ| > 0.8) between EPS components and microbial genera. Red and blue asterisks denote the genera with the highest number of significantly positive and negative correlations; (b) Correlation network. Nodes: Taxa grouped by interaction type (red: positive-correlation specialists; green: MP-dependent regulators; blue: negative-correlation specialists). Edges: Positive (solid red) and negative (dashed blue) associations. Node width ∝ connectivity degree; edge thickness ∝ |ρ|.

### Changes in Functional Genes on MPs

In Figure 5, relative abundances of biosynthesis-related genes on MPs in the bay are generally higher than those in the reservoir, whereas the pattern is reversed for quorum sensing-related genes. For protein biosynthesis, the dominant genes in the reservoir and the bay were *xapD* (73.2–138.1 RPKM) and *glnA* (199.9–227.1 RPKM), respectively. For polysaccharide biosynthesis, no genes showed clear dominance in the reservoir, whereas the relative abundances of *glmS* (103.4–118.7 RPKM) and *eno* (67.8–100.2 RPKM) increased significantly in the bay. For quorum sensing, the *liv* operon genes played a crucial role in the reservoir, particularly for *livH* (103.4–163.4 RPKM). Moreover, the genes related to protein biosynthesis and quorum sensing exhibited differential relative abundances in response to different MPs. For protein biosynthesis, the relative abundance of *glnA* (129.1 RPKM) on PVC was the highest, whereas the total relative abundance of all key genes was the lowest on TM in the reservoir. In the bay, the genes on PE exhibited higher abundances, particularly for *metH* (135.0 RPKM). For quorum sensing, the relative abundance of *livH* (163.4 RPKM) on PS was the highest, whereas the total relative abundance of all key genes was the lowest on PVC. Notably, the relative abundance of *ddpD* was significantly upregulated on PP in the bay.

**Figure 5:**
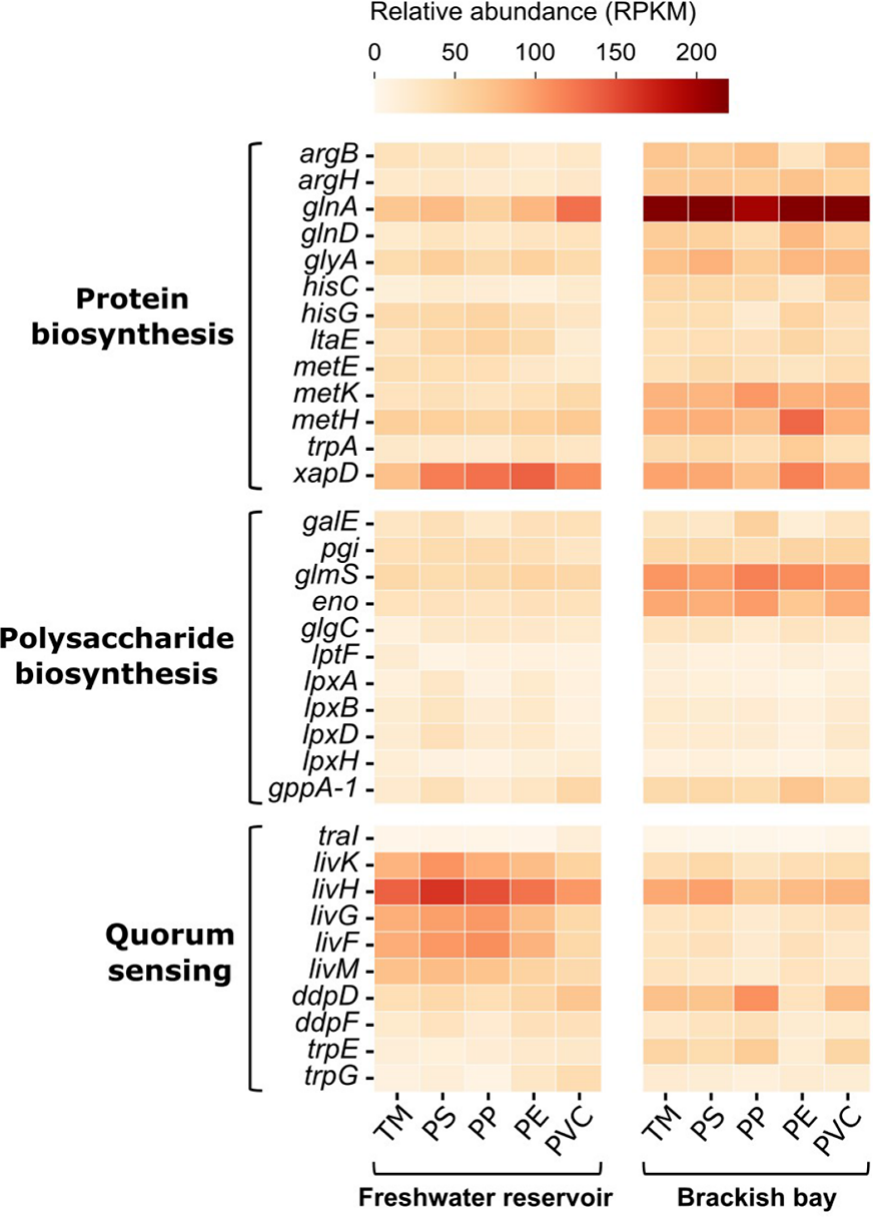
Relative abundances of functional genes related to protein biosynthesis, polysaccharide biosynthesis, and quorum sensing, respectively. Samples were collected on Day 60.

## Discussion

### Microbial Colonization Dynamics on Microplastics across Aquatic Ecosystems

MPs serve as selective substrates for microbial colonization, exhibiting distinct environmental patterning.^26^ MP-associated microbial communities showed elevated α-diversity (Shannon/Chao1 indices) compared to planktonic counterparts (Figure 1a,b), consistent with global plastisphere patterns.^27^ Microbial communities on MPs also exhibited significant segregation from those in the surrounding water (Figure S2a,b), further reinforcing the plastisphere’s role in shaping unique community structures. These findings highlight the importance of deciphering the ecological processes governing the selection and colonization of microorganisms on MPs in diverse urban aquatic ecosystems. In the reservoir, MP types significantly affected microbial community structure, particularly on PVC (Figure S2a). In contrast, microbial communities in the bay exhibited less differentiation across MP types, suggesting fewer substrate-specific effects (Figure S2b). These findings suggest that MP type plays a more pronounced role in shaping microbial colonization within higher-quality, less complex urban aquatic ecosystems (e.g., reservoirs), while the effects are mitigated in systems with higher environmental complexity (e.g., bays). Therefore, it is necessary to decipher the screening processes of various MPs on microorganisms in diverse urban aquatic ecosystems.

As to microbial communities, Proteobacteria, a well-known MP colonizer, consistently dominated the microbial communities on all MP types across both locations (Figure 1c,d), in line with findings from other plastisphere studies.^28^ Also, MPs facilitated the selective enrichment of indigenous microorganisms with typically low relative abundance in the surrounding water including Chloroflexi, Acidobacteria, and Nitrospirae,^29,30^ particularly in the bay. This phenomenon was more pronounced at the genus level, where community composition varied significantly between sites, except for *Novosphingobium*, which was consistently abundant in the bay. Besides, preferences for distinct MP types, indicated a strong association between microbial colonization behavior and MP materials. For example, at the phylum level, Actinobacteria, characterized by high relative abundance, were preferentially associated with PVC at both locations. As known of PE degraders via synthetic hydrolases,^31^ Actinobacteria may similarly degrade PVC through comparable enzymatic pathways, suggesting substrate versatility. At the genus level, *Pseudomonas*, a classical plastic-degrading bacterium,^32^ was not enriched on MPs, while *Acinetobacter* exhibited only short-term colonization on MPs in the bay. Temporal variations in microbial colonization further complicated the identification of dominant phyla and genera, highlighting the dynamic and MP-dependent nature of community assembly on MPs.

Thereafter, we investigated the ecological processes driving microbial colonization on MPs through community assembly, revealing significant variation in community assembly mechanisms across different aquatic environments. Both stochastic and deterministic processes were found to mediate microbial community on MPs at both sites (Figure 2a,b), consistent with observations of reservoir sediments.^33^ However, previous studies have pointed out contrasting roles of these processes depending on the aquatic system: stochastic processes were dominated in bays,^34^ while deterministic processes played a central role in lakes.^10^ In the reservoir, homogeneous selection (38.2%) was the most prominent process shaping microbial community on MPs, leading to stabilized taxonomic compositions. This finding aligns with Zhang et al. (2024), who observed that homogeneous selection as the primary process influencing MP-associated communities in freshwater lakes.^10^ The prevalence of homogeneous selection in the reservoir reflects consistent environmental conditions and limited variation. In contrast, the bay exhibited more variable environmental pressures, leading to an increased role of heterogeneous selection (27.9%) and a reduced influence of homogeneous selection (13.4%). Dispersal limitation (45.3%) dominated community assemblies on MPs in the bay, highlighting the dominance of stochastic forces in shaping microbial communities. Environmental disturbances in the bay, such as fluctuating water flow, amplified dispersal limitation, and other stochastic processes, as also noted by Zhang et al.^35^ These findings underscore the significant influence of environmental factors on the ecological processes governing microbial colonization on MPs. Stable environments, such as reservoirs, promote deterministic processes like homogeneous selection, whereas dynamic environments, such as bays, favor stochastic mechanisms, primarily dispersal limitation and heterogeneous selection.

Based on the results of environmental factors affecting microbial colonization on MPs, native microorganisms in the surrounding aquatic environment seemed to be the primary drivers of changes in MP-associated communities (Figure 2c). This can be attributed to the considerable divergence between native microbial community structures and those in the plastisphere. Environmental factors, such as salinity, dissolved oxygen, and pH, also played critical roles in shaping microbial communities.^36^ The physicochemical parameters of the water explained 36.5% of the variation in microbial composition. Among these, salinity showed the greatest fluctuations during the experiment and was likely the most influential factor. Nutrients, essential for microbial metabolism, further influenced microbial colonization and biofilm development. Wang et al. found that nutrients contributed 63% of the variations in microbial abundance on tire MPs.^27^ In this study, the bay, situated downstream, exhibited higher nutrient levels, leading to more abundant biofilms on MPs (Figure S1).

### Bacterial Succession Patterns at Temporal Scale

Exposure time played a critical role in shaping microbial community dynamics in the plastisphere, significantly influencing microbial succession.^9^ VPA results in Figure 2c indicate that exposure time accounted for nearly 40% of the variation in microbial succession. This highlights the necessity of further temporal-scale analyses to fully understand microbial diversity and richness over time. Rapid maturation of biofilms was observed at both sites, accompanied by an increase in diversity and richness, particularly in the bay. This temporal-scale difference was further confirmed by PERMANOVA analysis (*R* ^2^ = 0.44, *p* < 0.001), while NMDS analysis showed that the temporal succession of microbial communities on various MPs varied significantly. However, the effect of MP types was weakened in the complex urban system.

Microbial communities on MPs exhibited distinct temporal succession, characterized by shifts between early and late colonizers throughout the colonization process.^35^ Alphaproteobacteria, recognized as primary colonizers in aquatic environments, played a key role during the initial exposure phase.^35^ At both sites, genera such as *Sphingorhabdus*, *Novosphingobium*, and *Sphingomonas* were among early colonizers, exhibiting rapid growth on specific MPs (*Sphingorhabdus* on PS and PVC in the reservoir, *Novosphingobium* and *Sphingomonas* on all MPs). Additionally, *Aquabacterium* and *Hydrogenophaga* were identified as the early colonizers on PVC in the reservoir; while in the bay, *Hymenobacter*, *Massilia*, *Deinococcus*, and *Exiguobacterium* rapidly colonized the surfaces of all MPs. Notably, *Acinetobacter*, *Sphingobium* and *Aquabacterium* exhibited similar growth patterns on TM. Although these genera are less frequently reported as primary colonizers, it is hypothesized that their strong adaptability to MPs, coupled with responsiveness to environmental changes, enhances their capacity for early colonizing MPs. However, the relative abundances of these early colonizers declined significantly over time, indicating that while MPs selectively enrich certain bacterial populations in the short term, these communities are not stable. Early colonizers were eventually replaced by late colonizers, reflecting the dynamic nature of microbial communities on MPs. In the reservoir, the relative abundances of *Fimbriiglobus* and *Aggregatilinea* increased over time due to their polysaccharide hydrolysis potential^37^ and strong biodegradation capacity,^38^ may benefit from utilizing EPS secreted by early colonizers. In contrast, the bay exhibited greater diversity among late colonizers, including *Phycicoccus*, *Litoritinea*, and *Nitrospira*. Among these, *Nitrospira*, a nitrifying bacterium with the MP degradation potential,^39^ demonstrated stable colonization over time, likely due to the nitrogen-rich conditions in the bay. Yet, the colonization mechanisms of *Phycicoccus* and *Litoritinea* remain unclear. Overall, PVC in the reservoir and TM in the bay exhibited the most pronounced microbial succession at the genus level. Successional processes also varied across MP types, particularly among early colonizers in the reservoir. Furthermore, surface denaturation of MPs and interactions between microorganisms significantly influence microbial community succession, presenting a challenge for analyzing microbial community structures over time.

### Insights into EPS Secretion Behavior of Microorganisms on MPs

MPs serve a conducive environment for microbial colonization, stimulating the secretion of EPS and the biofilm formation.^6^ VPA showed that nutrients (24.0%) and physicochemical parameters (19.4%) played a regulatory role in bacterial EPS secretion (Figure 3c), leading to a significantly higher EPS concentration on MPs in the bay compared to the reservoir during the same exposure period (Figure 3a,b). The nutrient-rich environment in the bay enhanced microbial respiration and reproduction, thereby promoting EPS secretion. Moreover, the bay’s proximity to the ocean led to high salinity, which induced environmental stress and further boosted EPS production as a microbial stress response.^11,40^ Dissolved organic matter was another key factor influencing EPS secretion, as it is ubiquitously present in urban aquatic ecosystems and provides available substrates (e.g., sugars, amino acids, and organic acids) that promote microbial growth. Previous studies have shown that DOM enhances EPS production. For example, Liu et al. used dissolved organic matter to cultivate algal-bacterial granular sludge, observing increased tightly bound EPS and more active amino acid metabolism.^41^ Similarly, Yang et al. found that dissolved organic matter promoted colony formation by *Microcystis-*associated communities and enhanced EPS secretion.^42^ Although this study focuses on microbial-EPS-MP interactions rather than broader environmental conditions, it is evident that these external factors significantly influence microbial dynamics. Future research will explore their deeper impact on EPS production.

EPS composition analysis showed that the humic acid levels increased significantly on all MPs in the bay, driven by the activities of Acidobacteria and Actinobacteria. These taxa facilitated the humification of organic matter, leading to the accumulation of humic acids on MP surfaces.^30^ Proteins and polysaccharides, crucial EPS contents, also played various roles in biofilm formation. Proteins enhanced cell attachment and flocs formation, while polysaccharides formed network structures for cell colonization.^43^ A high protein-to-polysaccharide ratio typically promoted microbial accumulation and aggregation.^44^ In this study, the protein-to-polysaccharide ratios were higher in the bay, where polluted environments stimulated more intensive protein secretion on MPs to enhance microbial adaptation.

EPS secretion showed variation across MP types: higher EPS content was observed on TM and PE in the reservoir and on PP and PE in the bay. During exposure to different aquatic environments, EPS secretion was more active on PE and less active on PS, consistent with previous studies.^45^ Moreover, under stress conditions, microorganisms tend to secrete protein-rich EPS to adapt to unfavorable environments. This was evident from the higher protein-to-polysaccharide ratios on TM and PVC in the reservoir, with PVC showing the highest levels compared to other MPs in the bay due to its greater chemical toxicity. Capolupo et al. found that leachate from PVC and TM exhibited greater chemical toxicity relative to other MPs.^46^ Additionally, the protein-to-polysaccharide ratios were above 1.0 only on PVC in most cases. Although such ratios are typical in other systems, such as wastewater,^47^ discrepancies exist in different studies regarding EPS secretion on MPs,^45^ emphasizing the need for further research.

Biofilm formation and EPS production on MPs developed with prolonged exposure time. Given the close relationships between EPS and microbial growth, this study attempted to quantitatively or qualitatively analyze how EPS concentrations could link with microbial colonization on MPs. Such relationships have previously been observed, for instance, Wang et al. found similar trends in bacterial density and EPS contents on MPs.^48^ Correspondingly, similar variation trends were identified between microbial α-diversity and total EPS concentrations on MPs (particularly TM, PS, and PE) in the bay ecosystem (Tables S1 and S3). Here, we hypothesize preliminarily that the total EPS concentration might qualitatively predict the microbial α-diversity trends within the specific plastisphere, even though with varied accuracy depending on urban aquatic ecosystems and MP types. The application of EPS components, especially proteins, as biomass indicators, aligns with prior studies. Kleiner et al. quantified microbial biomass via macroproteomic protein abundance measurements,^49^ and Li et al. similarly used protein concentration to quantify the biomass on the electrode surfaces.^50^ This method displayed advantages over traditional biomass determination methods, such as adenosine triphosphate determination and scanning electron microscope, in terms of higher efficiency and reduced cost. Our study found that the predictive effect of proteins was generally consistent with total EPS concentration, whereas the predictive abilities of polysaccharides and humic acids were unstable and lacked sufficient theoretical support from relevant studies. Due to the complexity of microbial metabolic activities in the plastisphere, EPS secretion is regulated by various biotic and abiotic factors. Therefore, further validation of underlying mechanisms is required.

Microbial community structure on MPs accounted for 27.3% of the total variance in EPS components (Figure 3c). Phylum-level analysis indicated that the predominant phyla affecting the variations of EPS secretion on MPs were: Acidobacteria > Nitrospirae > Planctomycetes. Different phyla exhibited component-specific effects. Acidobacteria has been reported to secrete EPS containing a large number of unique polysaccharides,^51^ Nitrospirae abundance correlated positively with humic-like substances,^52^ and Planctomycetes secreted the compact extracellular proteins associated with biofilm formation.^53^ Genus-level correlation analysis (Figure 4) further revealed that the correlations between EPS components and different genera varied across MP types. For example, polysaccharides and humic acids showed intricate relationships with genera on TM, while humic acids correlated with genera on PE and PVC. Correlation patterns were particularly diverse on PS, but relatively simple on PP. Some genera (e.g., *Robiginitialea*, *Nitrospira*, and *Aquirufa*) showed significantly positive correlations with EPS components, while others such as *Fimbriiglobus*, *Terrimicrobium*, and *Legionella* exhibited significantly negative correlations with EPS components. Specifically, *Robiginitialea* was the most positively correlated with EPS components across MPs, though its overall impact was likely limited due to relatively low abundance at both sites. For *Fimbriiglobus*, negative correlations with polysaccharides observed on TM, PS, and PE MPs were likely attributed to its known polysaccharide-degrading capability (e.g., xylan, laminarin, lichenan, and chitin).^37^

Moreover, some studies have reported the relationships between EPS components and some genera (e.g., *Nitrospira* and *Terrimicrobium*), e.g., Yang et al. found enhanced EPS secretion where *Nitrospira* was the dominant genus.^54^ Correspondingly, our results indicated *Nitrospira* was significantly positively correlated with protein and polysaccharides on TM and PS. Conversely, *Terrimicrobium*, an anaerobic, carbohydrate-fermenter, potentially consumes EPS substrates under anoxic/anaerobic conditions,^55^ explaining its negative correlation with EPS components. Despite these findings, there is still a paucity of research examining the interaction mechanisms between genera and EPS components.

### Investigation of EPS Secretion Mechanisms via Metagenomic Sequencing

Metagenomic sequencing was applied to investigate the mechanisms of EPS secretion on MPs. Key genes directly regulating EPS secretion were identified, providing insight into microbial metabolic responses in the plastisphere (Figure 5). The abundance of biosynthesis-related genes increased under elevated environmental stress and bacterial proliferation, consistent with the higher concentrations of proteins and polysaccharides observed in the bay compared to the reservoir.

For protein biosynthesis, predominant genes varied between environments. In the reservoir, *xapD*, involved in purine nucleoside metabolism, was associated with the transmembrane transport of intracellular compounds and modulated the transport process of protein.^56^ In the bay, *glnA*, associated with glutamine synthesis, served as the precursor in protein biosynthesis. Other amino acid-related genes, such as *metH*, *metK* and *metE* (methionine), *glyA* (serine and glycine), *ltaE* (lysine), *argH* and *argB* (arginine), *hisG* and *hisC* (histidine), promoted the synthesis of dipeptides and polypeptides as protein precursors, ultimately boosting EPS secretion.^57^ On PVC in the reservoir, *glnA* (129.1 RPKM) confirmed that bacteria tended to biosynthesize protein. Moreover, the protein on PE might be enriched in methionine by the high relative abundance of *metH* (135.0 RPKM) in the bay. These findings suggest that bacteria exposed to elevated environmental stress may exhibit MP-type-specific tendencies in amino acid synthesis and protein production.

For polysaccharide biosynthesis, *glmS*, associated with peptidoglycan and lipopolysaccharide synthesis, and *eno* related to polysaccharide precursors synthesis, were significantly upregulated in the plastisphere in the bay. The upregulation of *glmS* suggested the enrichment of lipopolysaccharide in the carbohydrate composition on MPs.^58^ The upregulation of *eno* enhanced the glycolysis on MPs, ultimately promoting the polysaccharide biosynthesis and EPS metabolism.^59^

Quorum sensing regulated the microbial metabolism by providing energy and substrates necessary for EPS secretion as well. The gene *trpE* related to metabolite synthesis and the quorum sensing signaling,^60^ and *ddpD* involved in dipeptide transport,^61^ both exhibited higher relative abundance in the bay, particularly on PP. Conversely, the *liv* operon genes responsible for amino acid uptake for growth and reproduction,^62^ significantly decreased the nutrient-rich bay, likely because bacteria did not need to self-regulate to adapt to the aquatic environment. Limited research currently addresses indirect factors affecting EPS biosynthesis on MPs. For instance, unexplained biosynthetic phenomena could be related to other microbial activities in complex natural environments. A previous study showed that the “cell motility” pathway resulted in more active microbial movement on MPs, leading to a more dispersed distribution of microorganisms and EPS.^63^ In the future, more attention should be directed toward the indirect pathways that impact EPS distribution.

## Environmental Implications

Plastisphere, as the colonizing environment, enriched microorganisms in various urban aquatic ecosystems. During biofilm formation, EPS serves as essential mediators of interaction between microorganisms and the plastisphere, influencing microbial adhesion, aggregation, and activity. With EPS as a new perspective, this study comprehensively investigated the microbial-EPS-MP interactions, providing new insights into the ecological implications of MPs. The plastisphere fostered the microbial community structure that differed from that in the aquatic environment, thereby rendering MPs as microorganism carriers with potential biological security risks. Specific phyla (e.g., Acidobacteria, Nitrospirae, and Planctomycetes) and genera (e.g., *Robiginitialea* and *Fimbriiglobus*) were identified as key contributors to variations in EPS components across different MPs. Mechanistically, the plastisphere-regulated functional genes, involved in protein biosynthesis (e.g., *glnA* and *xapD*), polysaccharide biosynthesis (e.g., *glmS* and *eno*), and biofilm formation (e.g., *liv* operon genes), played pivotal roles in mediating microbial EPS secretion. These interactions were further influenced by environmental conditions, highlighting the dynamic interplay between microbial processes, MPs, and their surrounding ecosystems. For instance, in high-water quality urban aquatic ecosystems like the reservoir, the impact of MP types (e.g., PVC) on microbial community structure was further amplified. In contrast, environments under high selective pressures, such as the bay, promoted more intense EPS secretion, particularly in the form of protein-dominant EPS secretion. PVC offered a distinctive colonization plastisphere for microorganisms in the aquatic environment. These findings underline the importance of understanding MP-specific plastisphere dynamics and their environmental consequences. The outcomes of this study have far-reaching implications for ecological research and environmental management. Practical applications include the development of EPS-regulated advanced MP removal technologies, the construction of biofilm-microbial characteristic databases, and the establishment of microbial diversity-MP type/concentration–response models. These will provide critical references and data support for ecological remediation and risk assessment of MP pollution. Moreover, given the high diversity of urban aquatic ecosystems, the site selection restricted the broader applicability of the conclusions. Future studies should pay more attention to other urban aquatic ecosystems.

## Supplementary Material



## Notes

Research site, sampling, and environmental parameter measurement, microplastic incubation experiment setup, EPS extraction and composition analysis, DNA extraction, 16S rDNA amplicon and metagenomic shotgun sequencing, statistical analysis, data of α-diversity, data of water quality at each research site, data of EPS component concentration and protein-to-polysaccharide ratios on MPs, experimental procedure, morphological characteristics of each MP sample, and figure of the variations of water quality (PDF)

## Abbreviations

MPs: microplastics
PE: polyurethane
PP: polypropylene
PS: polystyrene
PVC: polyvinyl chloride
TM: tire material
GB: glass beads
EPS: extracellular polymeric substances
NMDS: nonmetric multidimensional scaling
VPA: variation partitioning analysis
PERMANOVA: permutational multivariate analysis of variance

## References

[1] Mamun A. A., Prasetya T. A. E., Dewi I. R., Ahmad M. (2023). Microplastics in human
food chains: Food becoming a threat to health safety. Sci. Total Environ..

[2] Nava V., Chandra S., Aherne J., Alfonso M. B., Antao-Geraldes A. M., Attermeyer K., Bao R., Bartrons M., Berger S. A., Biernaczyk M., Bissen R., Brookes J. D., Brown D., Canedo-Argueelles M., Canle M., Capelli C., Carballeira R., Cereijo J. L., Chawchai S., Christensen S. T., Christoffersen K. S., de Eyto E., Delgado J., Dornan T. N., Doubek J. P., Dusaucy J., Erina O., Ersoy Z., Feuchtmayr H., Frezzotti M. L., Galafassi S., Gateuille D., Goncalves V., Grossart H.-P., Hamilton D. P., Harris T. D., Kangur K., Kankilic G. B., Kessler R., Kiel C., Krynak E. M., Leiva-Presa A., Lepori F., Matias M. G., Matsuzaki S.-I. S., McElarney Y., Messyasz B., Mitchell M., Mlambo M. C., Motitsoe S. N., Nandini S., Orlandi V., Owens C., Ozkundakci D., Pinnow S., Pociecha A., Raposeiro P. M., Room E.-I., Rotta F., Salmaso N., Sarma S. S. S., Sartirana D., Scordo F., Sibomana C., Siewert D., Stepanowska K., Tavsanoglu U. N., Tereshina M., Thompson J., Tolotti M., Valois A., Verburg P., Welsh B., Wesolek B., Weyhenmeyer G. A., Wu N., Zawisza E., Zink L., Leoni B. (2023). Plastic debris in lakes
and reservoirs. Nature.

[3] Li Y., Deng Y., Hu C., Li D., Zhang J., Zhou N. (2024). Microplastic pollution in urban rivers
within China’s Danxia
landforms: Spatial distribution characteristics, migration, and risk
assessment. Sci. Total Environ..

[4] Tang N., Yu Y., Cai L., Tan X., Zhang L., Huang Y., Li B., Peng J., Xu X. (2022). Distribution Characteristics and
Source Analysis of Microplastics in Urban Freshwater Lakes: A Case
Study in Songshan Lake of Dongguan, China. Water.

[5] Koutnik V. S., Leonard J., Alkidim S., DePrima F. J., Ravi S., Hoek E. M. V., Mohanty S. K. (2021). Distribution
of microplastics in
soil and freshwater environments: Global analysis and framework for
transport modeling. Environ. Pollut..

[6] Gonzalez-Menendez C., Sol D., Laca A., Laca A., Diaz M. (2024). Interrelation between
extracellular polymer substances (EPSs) and MPs in an MBR. Journal of Environmental Chemical Engineering.

[7] He S., Jia M., Xiang Y., Song B., Xiong W., Cao J., Peng H., Yang Y., Wang W., Yang Z., Zeng G. (2022). Biofilm on
microplastics in aqueous environment: Physicochemical
properties and environmental implications. Journal
of Hazardous Materials.

[8] Amaral-Zettler L. A., Zettler E. R., Mincer T. J. (2020). Ecology of the plastisphere. Nature Reviews Microbiology.

[9] Wang T., Lu F., Yang C., Wang C., Liao Y., Mkuye R., Deng Y. (2024). Exploring
changes in microplastic-associated bacterial communities
with time, location, and polymer type in Liusha Bay, China. Marine Environmental Research.

[10] Zhang W., Bhagwat G., Palanisami T., Liang S., Wan W., Yang Y. (2024). Lacustrine plastisphere:
Distinct succession and assembly processes
of prokaryotic and eukaryotic communities and role of site, time,
and polymer types. Water Res..

[11] Feng C., Lotti T., Canziani R., Lin Y., Tagliabue C., Malpei F. (2021). Extracellular biopolymers recovered
as raw biomaterials
from waste granular sludge and potential applications: A critical
review. Sci. Total Environ..

[12] Gong X., Ge Z., Ma Z., Li Y., Huang D., Zhang J. (2023). Effect of
different size microplastic particles on the construction of algal-bacterial
biofilms and microbial communities. Journal
of Environmental Management.

[13] Hung C.-M., Chen C.-W., Huang C.-P., Hsieh S.-L., Dong C.-D. (2022). Ecological
responses of coral reef to polyethylene microplastics in community
structure and extracellular polymeric substances. Environ. Pollut..

[14] APHA . Standard Methods For the Examination of Water and Wastewater, 21st ed.; American Public Health Association: Washington, DC, 2005.

[15] Feng C., Lotti T., Lin Y., Malpei F. (2019). Extracellular polymeric
substances extraction and recovery from anammox granules: Evaluation
of methods and protocol development. Chemical
Engineering Journal.

[16] DuBois M., Gilles K. A., Hamilton J. K., Rebers P. A., Smith F. (1956). Colorimetric
Method for Determination of Sugars and Related Substances. Anal. Chem..

[17] Frolund B., Griebe T., Nielsen P. H. (1995). Enzymatic activity in the activated-sludge
floc matrix. Appl. Microbiol. Biotechnol..

[18] Li D., Liu C.-M., Luo R., Sadakane K., Lam T.-W. (2015). MEGAHIT:
an ultra-fast single-node solution for large and complex metagenomics
assembly via succinct *de Bruijn* graph. Bioinformatics.

[19] Hyatt D., Chen G.-L., LoCascio P. F., Land M. L., Larimer F. W., Hauser L. J. (2010). Prodigal: prokaryotic
gene recognition and translation
initiation site identification. BMC Bioinf..

[20] Wood D. E., Salzberg S. L. (2014). Kraken: ultrafast metagenomic sequence
classification
using exact alignments. Genome Biol..

[21] Lu J., Breitwieser F. P., Thielen P., Salzberg S. L. (2017). Bracken:
estimating
species abundance in metagenomics data. Peerj
Computer Science.

[22] Zhou Z., Tran P. Q., Breister A. M., Liu Y., Kieft K., Cowley E. S., Karaoz U., Anantharaman K. (2022). METABOLIC:
high-throughput profiling of microbial genomes for functional traits,
metabolism, biogeochemistry, and community-scale functional networks. Microbiome.

[23] Oksanen, A. J. ; Blanchet, F. G. ; Friendly, M. ; Kindt, R. ; Legendre, P. ; Mcglinn, D. ; Minchin, P. R. ; Hara, R. B. O. ; Simpson, G. L. ; Solymos, P. ; Stevens, M. H. H. ; Szoecs, E. ; Vegan. In Encyclopedia of Food and Agricultural Ethics; Kaplan, D. M. , Ed.; Springer Netherlands: Dordrecht, 2019; pp 2395–2396.

[24] Ning D., Yuan M., Wu L., Zhang Y., Guo X., Zhou X., Yang Y., Arkin A. P., Firestone M. K., Zhou J. (2020). A quantitative framework
reveals ecological drivers of grassland
microbial community assembly in response to warming. Nat. Commun..

[25] Lai J., Zou Y., Zhang J., Peres-Neto P. R. (2022). Generalizing hierarchical and variation
partitioning in multiple regression and canonical analyses using the
rdacca.hp R package. Methods in Ecology and
Evolution.

[26] Zhou Z., Tang J., Tang K., An M., Liu Z., Wu Z., Cao X., He C. (2024). Selective enrichment of bacteria
and antibiotic resistance genes in microplastic biofilms and their
potential hazards in coral reef ecosystems. Chemosphere.

[27] Wang L., Luo Z., Zhen Z., Yan Y., Yan C., Ma X., Sun L., Wang M., Zhou X., Hu A. (2020). Bacterial community
colonization on tire microplastics in typical urban water environments
and associated impacting factors. Environ. Pollut..

[28] Pan X., Lin L., Cao X., Jing Z., Dong L., Zhai W. (2024). Response of
microbial communities and biogeochemical cycling functions to sediment
physicochemical properties and microplastic pollution under damming
and water diversion projects. Sci. Total Environ..

[29] Li W., Wang Z., Li W., Li Z. (2022). Impacts of microplastics
addition on sediment environmental properties, enzymatic activities
and bacterial diversity. Chemosphere.

[30] Ye T., Huang M., Wang Y., Yang A., Xu H. (2024). Humic substance
mitigated the microplastic-induced inhibition of hydroxyl radical
production in riparian sediment. Journal of
Hazardous Materials.

[31] Yi M., Zhou S., Zhang L., Ding S. (2021). The effects of three
different microplastics on enzyme activities and microbial communities
in soil. Water Environment Research.

[32] Dong X., Zhu L., He Y., Li C., Li D. (2023). Salinity significantly
reduces plastic-degrading bacteria from rivers to oceans. Journal of Hazardous Materials.

[33] Li Y., Gao Y., Zhang W., Wang C., Wang P., Niu L., Wu H. (2019). Homogeneous
selection dominates the microbial community assembly
in the sediment of the Three Gorges Reservoir. Sci. Total Environ..

[34] Sun Y., Zhang M., Duan C., Cao N., Jia W., Zhao Z., Ding C., Huang Y., Wang J. (2021). Contribution
of stochastic processes to the microbial community assembly on field-collected
microplastics. Environmental Microbiology.

[35] Zhang S.-J., Zeng Y.-H., Zhu J.-M., Cai Z.-H., Zhou J. (2022). The structure
and assembly mechanisms of plastisphere microbial community in natural
marine environment. Journal of Hazardous Materials.

[36] Zhou L., Wang M., Zhang S., Jiang H., Liu H., Chen X., Zhong L. (2024). Spatial distribution
of bacterial
communities driven by multiple environmental factors in sediment of
brackish channel catfish ponds in Eastern China. Aquaculture.

[37] Ravin N. V., Rakitin A. L., Ivanova A. A., Beletsky A. V., Kulichevskaya I. S., Mardanov A. V., Dedysh S. N. (2018). Genome Analysis of *Fimbriiglobus
ruber* SP5^T^, a Planctomycete with Confirmed Chitinolytic
Capability. Appl. Environ. Microbiol..

[38] Dong C.-D., Cheng J.-W., Chen C.-W., Huang C.-P., Hung C. M. (2023). Activation
of calcium peroxide by nitrogen and sulfur co-doped metal-free lignin
biochar for enhancing the removal of emerging organic contaminants
from waste activated sludge. Bioresour. Technol..

[39] Wu C., Ma Y., Wang D., Shan Y., Song X., Hu H., Ren X., Ma X., Cui J., Ma Y. (2022). Integrated microbiology
and metabolomics analysis reveal plastic mulch film residue affects
soil microorganisms and their metabolic functions. Journal of Hazardous Materials.

[40] Guo G., Tian F., Ding K., Yang F., Wang Y., Liu C., Wang C. (2023). Effect of
salinity on removal performance of anaerobic
membrane bioreactor treating azo dye wastewater. Appl. Biochem. Biotechnol..

[41] Liu Z., Duan Y., Hou Y., Zhang S., Wang J., Gao M., Zhang A., Liu Y. (2024). Evaluating the role of carbon sources
on the development of algal-bacterial granular sludge: From sludge
characteristics, extracellular polymer properties, quorum sensing,
and microbial communities. Journal of Cleaner
Production.

[42] Yang C., Ding M., Hou K., Feng J., Li X., Pan X., Yang C., Zhang X., Guo J., Dai X. (2024). Dissolved
organic matter, calcium ion and extracellular polymeric substances
on living associated bacteria of *Microcystis* colony
are crucial for unicellular *Microcystis* to efficiently
form colonies. Journal of Hazardous Materials.

[43] Flemming H.-C., van Hullebusch E. D., Little B. J., Neu T. R., Nielsen P. H., Seviour T., Stoodley P., Wingender J., Wuertz S. (2025). Microbial extracellular
polymeric substances in the
environment, technology and medicine. Nature
Reviews Microbiology.

[44] Chen H., Li A., Cui C., Ma F., Cui D., Zhao H., Wang Q., Ni B., Yang J. (2019). AHL-mediated
quorum
sensing regulates the variations of microbial community and sludge
properties of aerobic granular sludge under low organic loading. Environ. Int..

[45] Deng H., Fu Q., Li D., Zhang Y., He J., Feng D., Zhao Y., Du G., Yu H., Ge C. (2021). Microplastic-associated
biofilm in an intensive mariculture pond: Temporal dynamics of microbial
communities, extracellular polymeric substances and impacts on microplastics
properties. Journal of Cleaner Production.

[46] Capolupo M., Sorensen L., Jayasena K. D. R., Booth A. M., Fabbri E. (2020). Chemical composition
and ecotoxicity of plastic and car tire rubber leachates to aquatic
organisms. Water Res..

[47] Sodhi V., Bansal A., Jha M. K. (2022). Effect
of extracellular polymeric
compositions on in-situ sludge minimization performance of upgraded
activated sludge treatment for industrial wastewater. Journal of Environmental Management.

[48] Wang H., Xu K., Wang J., Feng C., Chen Y., Shi J., Ding Y., Deng C., Liu X. (2023). Microplastic biofilm:
An important microniche that may accelerate the spread of antibiotic
resistance genes via natural transformation. Journal of Hazardous Materials.

[49] Kleiner M., Thorson E., Sharp C. E., Dong X., Liu D., Li C., Strous M. (2017). Assessing species biomass contributions
in microbial
communities via metaproteomics. Nat. Commun..

[50] Li X., Tian X., Yan X., Huo N., Wu X., Zhao F. (2023). Lumichrome from the photolytic riboflavin acts as an electron shuttle
in microbial photoelectrochemical systems. Bioelectrochemistry.

[51] Zhang J., Shi Q., Fan S., Zhang Y., Zhang M., Zhang J. (2021). Distinction
between Cr and other heavy-metal-resistant bacteria involved in C/N
cycling in contaminated soils of copper producing sites. Journal of Hazardous Materials.

[52] Ren J., Cheng W., Wan T., Wang M., Meng T., Lv T. (2019). Characteristics of
the extracellular polymeric substance composition
in an up-flow biological aerated filter reactor: The impacts of different
aeration rates and filter medium heights. Bioresour.
Technol..

[53] Tang L., Su C., Fan C., Cao L., Liang Z., Xu Y., Chen Z., Wang Q., Chen M. (2023). Metagenomic and extracellular
polymeric substances analysis reveals the mechanism of exogenous N-hexanoyl-L-homoserine
lactone in alleviating the inhibition of perfluorooctanoic acid on
anammox process. Bioresour. Technol..

[54] Yang Q., Zhong Y., Feng S.-W., Wen P., Wang H., Wu J., Yang S., Liang J.-L., Li D., Yang Q., Tam N. F. Y., Peng P. A. (2024). Temporal enrichment
of comammox *Nitrospira* and Ca. Nitrosocosmicus in
a coastal plastisphere. ISME J..

[55] Gao X., Zhang L., Liu J., Zhang Y., Peng Y. (2024). First application
of the novel anaerobic/aerobic/anoxic (AOA) process for advanced nutrient
removal in a wastewater treatment plant. Water
Res..

[56] Fujikawa T., Ogura Y., Ishigami K., Kawano Y., Nagamine M., Hayashi T., Inoue K. (2021). Unexpected
genomic features of high
current density-producing *Geobacter sulfurreducens* strain YM18. FEMS Microbiol. Lett..

[57] Wang Y.-H., Wu Y.-H., Luo L.-W., Wang Q., Tong X., Bai Y., Ni X.-Y., Wang H.-B., Chen G.-Q., Nozomu I., Chen Z., Hu H.-Y. (2021). Metagenomics
analysis of the key
functional genes related to biofouling aggravation of reverse osmosis
membranes after chlorine disinfection. Journal
of Hazardous Materials.

[58] Yeom J., Lee Y., Park W. (2012). Effects of non-ionic
solute stresses on biofilm formation
and lipopolysaccharide production in *Escherichia coli* O157:H7. Research in Microbiology.

[59] Ramos A., Boels I. C., de Vos W. M., Santos H. (2001). Relationship between
glycolysis and exopolysaccharide biosynthesis in *Lactococcus
lactis*. Appl. Environ. Microbiol..

[60] Han J., Xu W., Zhu J., Su X., Fu H., Xiao X., Dong F., Chen C., Lin H., Sun F. (2024). Efficient
nitrogen removal and stable operation of a dynamic membrane bioreactor
(DMBR) for landfill leachate treatment. Chemical
Engineering Journal.

[61] Fang S., Wu Q., Wei Z., Cao W., Cheng S., Wang D., He C., Zhao Y., Cao J., Luo J. (2024). Methylisothiazolinone
modulates community assembly and improves syntrophic cooperation via
adaptive evolution during sludge anaerobic digestion. Chemical Engineering Journal.

[62] Nazos P. M., Antonucci T. K., Landick R., Oxender D. L. (1986). Cloning and characterization
of livH, the structural gene encoding a component of the leucine transport
system in Escherichia coli. Journal of bacteriology.

[63] Bera P., Wasim A., Ghosh P. (2023). Interplay of cell motility and self-secreted
extracellular polymeric substance induced depletion effects on spatial
patterning in a growing microbial colony. Soft
Matter.

