# Supplementary material for: Microbial Dynamics on Different Microplastics in Coastal Urban Aquatic Ecosystems: The Critical Roles of Extracellular Polymeric Substances
Source: Environ Sci Technol. 2025 May 20;59(21):10554–66. doi: 10.1021/acs.est.5c03796 (PMC12139041; doi:10.1021/acs.est.5c03796)
Supplement: Supplementary file 1 [file es5c03796_si_001.pdf]

Supporting Information

Microbial dynamics on different microplastics in coastal urban aquatic ecosystems: the critical roles of extracellular polymeric substances

Cuijie Feng [a], Ziyan Liang [a], Xin Liao[b], Kairong Lin [a], Yujia Zhai [c], Gang Liu [d, e, *], Francesca Malpei [f], Anyi Hu [g, *]

[a] *Center for Water Resources and Environment, School of Civil Engineering, Sun Yat-sen University, Guangzhou 510275, PR China*

[b] *CAS Key Laboratory of Urban Pollutant Conversion, Institute of Urban Environment, Chinese Academy of Science, Xiamen 361021, PR China*

[c] *State Key Joint Laboratory of Environment Simulation and Pollution Control, School of Environment, Beijing Normal University, Beijing 100875, PR China*

[d] *Key Lab of Aquatic Chemistry, State Key Lab of Regional Environment, Research Centre for Eco-Environmental Sciences, Chinese Academy of Sciences, Beijing, 100085, China*

[e] *Sanitary engineering, Department of Water management, Delft University of Technology, Delft 2628 CN, the Netherlands*

[f] *Department of Civil and Environmental Engineering, Politecnico di Milano, Milan 20133, Italy*

[g] *Carbon Neutral Innovation Research Center and Fujian Key Laboratory of Marine Carbon Sequestration, Xiamen University, Xiamen 361105, PR China*

[*] To whom correspondence should be addressed. **Email:** gliu@rcees.ac.cn, g.liu-1@tudelft.nl (G. Liu); ayhu@xmu.edu.cn (A. Hu)

Summary: 7 pages, 1 table, 2 figures

Contents

Texts

Text S1. Water Quality at Each Research Site

Table

Table S1. The α-diversity of microbial communities on MPs

Figure

Figure S1. The variations of physicochemical parameters for (a) the reservoir and (c) the bay during the experiment. The variations of nutrients for (b) the reservoir and (d) the bay. EC, electric conductivity; DO, dissolved oxygen; T, temperature; SAL, salinity; NH_4^+-N, ammonia; NO_2^--N, nitrite; NO_3^--N, nitrate; PO_4^{3-}-P, phosphate.

Figure S2. Comparative analysis of β-diversity (NMDS) across aquatic environments: (a) reservoir and (b) bay.

Text S1. Water Quality at Each Research Site

As presented in Figure S1a, b, both the reservoir and the bay were weakly alkaline, with the reservoir generally more alkaline. Dissolved oxygen of the reservoir exhibited a decreasing trend, but consistently remained higher than the bay. The salinity of the reservoir remained below 0.05‰, whereas the bay consistently exhibited higher salinity levels. By day 90, the bay was characterized as brackish water with a salinity of 9.31‰. Additionally, nutrient levels in the bay were higher than those in the reservoir (Figure Sc, d). Ammonia levels in the reservoir were nearly 0 $mg\cdot L^{-1}$, while in the bay, they showed significant variation, with a maximum difference of 1.008 $mg\cdot L^{-1}$ between the two sites. Nitrite concentrations were also close to 0 $mg\cdot L^{-1}$ in the reservoir, and did not exceed 0.1 $mg\cdot L^{-1}$ in the bay. Nitrate levels in the reservoir initially increased and then decreased, surpassing the bay only on day 60. Phosphate concentrations in the bay remained within the range of 0.1-0.2 $mg\cdot L^{-1}$ (except on 30 day), while they exhibited minor fluctuations in the reservoir.

Table S1. The α-diversity of microbial communities on MPs

Site	Type	Time (d)	Shannon	Chao1
Reservoir	Water	0	2.83	1351.56
		15	3.23	1029.05
		30	3.58	1217.64
		60	3.79	1457.33
		90	4.26	1432.32
	GB	90	6.13	1879.28
	TM	15	5.48	3209.46
		30	5.03	2529.76
		60	5.32	2308.51
		90	5.71	3076.21
	PS	15	5.03	1536.70
		30	5.97	2421.09
		60	6.01	3121.71
		90	5.66	2482.76
	PP	90	5.76	2291.15
	PE	30	5.52	1677.91
		60	5.81	2480.67
		90	5.81	2459.99
	PVC	15	3.68	1691.08
		30	4.86	2235.14
		60	5.25	2388.51
		90	5.99	3068.99
Bay	Water	0	4.54	3192.65
		15	4.99	3863.56
		30	6.15	5973.74
		60	5.58	4283.99
		90	5.24	3083.67
	GB	15	3.33	990.00
		30	5.44	5437.84
		60	6.64	7181.43
		90	5.88	5363.11
	TM	15	3.45	1081.53
		30	6.28	5998.39
		60	6.75	7016.08
		90	5.83	5219.29
	PS	15	3.32	1524.30
		30	6.62	6822.99
		60	6.90	7521.90
		90	6.09	5987.90
	PP	15	3.83	1438.13
		30	5.53	5446.43
		60	6.62	7301.49
		90	6.75	6324.80

PE	15	3.80	1294.97
	30	6.48	6311.96
	60	5.85	5629.36
	90	6.71	6637.43
PVC	15	3.20	1135.14
	30	5.98	5889.82
	60	6.53	7272.46
	90	6.13	5559.00

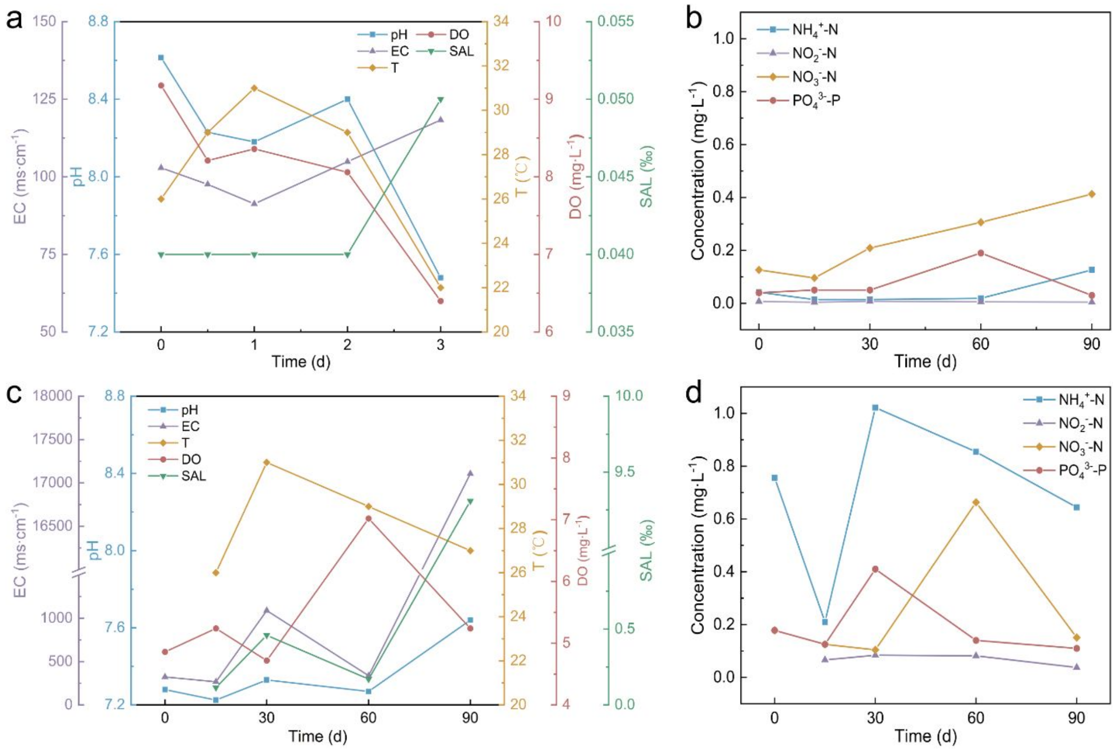

Figure S1. The variations of physicochemical parameters for (a) the reservoir and (c) the bay during the experiment. The variations of nutrients for (b) the reservoir and (d) the bay. EC, electric conductivity; DO, dissolved oxygen; T, temperature; SAL, salinity; NH_4^+-N, ammonia; NO_2^--N, nitrite; NO_3^--N, nitrate; PO_4^{3-}-P, phosphate.

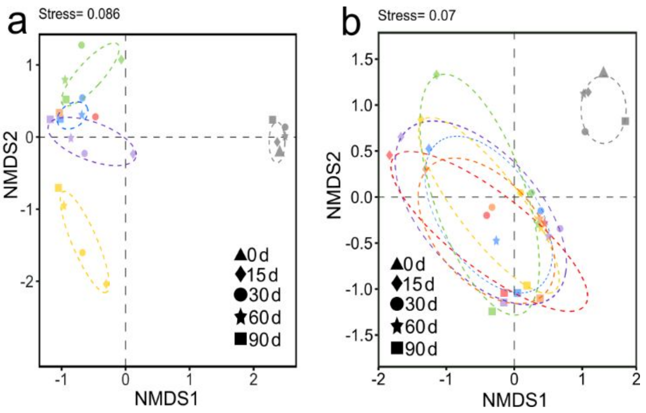

Figure S2. Comparative analysis of β-diversity (NMDS) across aquatic environments: (a) reservoir and (b) bay.
